# Recrystallization and Second-Phase Precipitation in Nb-V Microalloyed Steels: A Thermal Simulation Study

**DOI:** 10.3390/ma18133069

**Published:** 2025-06-27

**Authors:** Qilin Ma, Shubiao Yin, Chengjia Shang, Qingyou Liu, Ba Li, Shujun Jia

**Affiliations:** 1 Central Iron and Steel Research Institute Co., Ltd., Beijing 100081, China; maqilin0812@163.com (Q.M.); yue06282714@163.com (Q.L.); balicugb@sina.com (B.L.); 2 Collaborative Innovation Center for Steel Commonality, University of Science and Technology Beijing, Beijing 100083, China; 18332260911@163.com; 3 Faculty of Metallurgical and Energy Engineering, Kunming University of Science and Technology, Kunming 650093, China; yinshubiao@kust.edu.cn

**Keywords:** Nb-V microalloying, recrystallization behavior, second-phase precipitation, thermodynamic and kinetic calculations, precipitation kinetics

## Abstract

This study investigates the relationship between recrystallization behavior and second-phase precipitation in Nb-V microalloyed steel during the rough rolling stage through thermal simulation experiments. The effects of deformation amount and temperature on austenite recrystallization were analyzed, alongside thermodynamic and kinetic calculations to assess the influence of Nb-V microalloying on second-phase precipitation. The results show that both the deformation amount and temperature significantly affect recrystallization, with Nb-V steel exhibiting more pronounced grain refinement compared to Nb steel. Significant differences in the type, morphology, and size distribution of second-phase precipitates were observed, with Nb-V steel primarily precipitating (Nb, V)C, while Nb steel only precipitates NbC. The average size of second-phase particles in Nb-V steel (10.60 nm) is smaller and more uniformly dispersed than in Nb steel (33.85 nm). Thermodynamic and kinetic analyses indicate that Nb-V microalloying accelerates second-phase precipitation kinetics. Moreover, second-phase particles hinder grain-boundary motion during recrystallization, with their effect surpassing that of Nb and V solid-solution atoms. These findings enhance the understanding of Nb-V composites in refining austenite grain size and promoting second-phase precipitation, providing valuable insights into the design and processing of high-performance microalloyed steels.

## 1. Introduction

Low-carbon microalloyed steels are widely used in critical sectors such as automotive manufacturing (for lightweight chassis and crash-resistant components), construction (for seismic-resistant structural beams), energy infrastructure (offshore wind turbine towers and oil/gas pipelines), and aerospace (high-temperature resistant fasteners), owing to their excellent toughness, weldability, and formability [1,2,3]. Microalloying technology is used to enhance the toughness matching of steel by adding elements such as Nb, V, and Ti and optimizing their ratios [4,5]. Among them, Nb, as a key microalloying element, plays an important role in refining austenite grain, improving precipitation strengthening, and enhancing hardenability. However, under the traditional controlled rolling process, high Nb mild steel often suffers from uneven organization and mixed crystals, which seriously affect the stability of its properties [6,7]. In recent years, the development of HTP (High Temperature Processing) technology for the application of high Nb pipeline steel has provided a new way of thinking. The core of HTP technology is to increase the rolling temperature, i.e., the Nb in the austenite full solid solution, so as to increase the termination temperature of the austenite recrystallization, so that it is still rolling after the excellent heat-affected zone (HAZ) toughness [8]. Heating and holding above 1200 °C can ensure the full solid solution of Nb, increase the uncrystallized temperature [9,10,11], mediate precipitation strengthening in ferrite, and ultimately optimize the steel properties. Studies have shown that the solid solution and precipitation of high Nb in austenite play an important role in grain refinement, precipitation strengthening, and inhibition of austenite recrystallization [12,13,14]. However, too high Nb content may lead to coarse precipitation phases, and the relationship between coarse precipitation phases and recrystallization has not been clarified; it is thought that the addition of high Nb also has the problems of poor weldability and softening of welded joints [15,16,17]. Therefore, the optimized design of high Nb mild steel alone still faces many challenges.

In contrast to Nb, vanadium (V)—a strategic resource with abundant reserves in China—has been widely adopted in microalloyed steels due to mature industrial applications. The unique precipitation behavior of V contributes to its versatility: it enables interphase precipitation during γ→α transformation and intra-ferritic precipitation, both of which enhance strength through fine-scale (V, Nb)(C, N) dispersions. High solubility in austenite (>1.2 wt.% at 1180 °C) allows V to remain predominantly in solid solution above 1180 °C, resulting in weaker recrystallization inhibition compared to Nb. This property facilitates post-rolling grain coarsening control through tailored cooling schedules [18]. It is mainly precipitated and strengthened during cooling and phase transformation. In addition, every 0.1% increase in V can increase the yield strength of steel by about 200 MPa [19]. More importantly, V has the potential to improve welding properties: not only can it reduce the tendency of Weiss organization in the weld heat affected zone (HAZ) and promote the formation of intracrystalline ferrite but a large number of V-containing secondary phases precipitated from the ferrite phase zone is also conducive to improving the softening of welded joints [20,21,22].

At present, a large number of studies have been carried out on the hot deformation, recrystallization behavior, and precipitation strengthening mechanism of high Nb steels but the interaction between the recrystallization behavior of Nb-V composite microalloying and the second phase particles at the hot rolling stage has not been clarified. Prior studies on Nb-V steels via deformation dilatometry [23] revealed that plastic deformation in non-recrystallization regimes significantly accelerates both ferrite and bainite transformations, shifting CCT curves to higher temperatures. However, this acceleration diminishes at lower cooling rates (<5 °C/s), promoting polygonal ferrite formation while exacerbating microstructural heterogeneities. X Guo [24] investigated the effects of different quenching temperatures on the microstructure and mechanical properties of steel. The results showed that the quenching temperature should be controlled below 1100 °C and the holding time should not exceed 60 min to avoid abnormal grain growth. The thermodynamic calculations and experimental results have certain theoretical guidance significance for the practical industrial application of high-niobium-titanium steels. However, the study is only for the reheating stage of hot rolling, and the influence on the subsequent roughing and finishing organization and properties is not clear. Zhou Qi et al. [25] analyzed the microstructure evolution and precipitation behavior of Nb-V microalloyed steel during isothermal processing through thermal simulation experiments. The results showed that with the increase in holding time, the hardness of the test steel decreased, the precipitation phase particles coarsened, and the microstructure was transformed from martensite and bainite ferrite to granular bainite and polygonal ferrite. In the experiment on Nb-V composite microalloyed steel organization transformation, the precipitation phase changes in the study are more systematic and have a certain reference value and significance. However, this study for the second phase particle precipitation only stays in the stage of characterization, lacking further analysis and calculation. Zhang Ke et al. [26] explored the kinetic characteristics of Nb-V-Ti composite precipitation based on the thermodynamic calculation of the multivariate composite precipitation phase. However, these studies mainly focused on the post-rolling tissue evolution and precipitation thermodynamic analysis, yet there is still a lack of systematic studies on the austenite grain size evolution, recrystallization behavior, and precipitation mechanism during the deformation process of Nb-V composite pairs.

This study establishes a quantitative process–structure link in Nb/V-microalloyed pipeline steels during rough rolling, addressing two interdependent challenges:

To unravel how Nb-V microalloying modulates the competition between austenite recrystallization kinetics and (Nb,V)C precipitation behavior, under variable thermomechanical conditions (980–1050 °C, 15–30% reduction).

To determine whether refined precipitation kinetics (experimentally evidenced by shifted PTT curves) can counteract mixed grain formation induced by non-uniform recrystallization, ultimately enhancing strength–toughness synergy. In order to improve the composition design of high-performance pipeline steel and to provide a theoretical basis for engineering applications, it is suggested that Nb-V composites should be used in the design of high-performance pipeline steel. In summary, this study not only fills the gap in the research on the recrystallization behavior and second phase precipitation of Nb-V composite microalloyed steel but it also provides systematic theoretical guidance and a practical basis for the composition design, process optimization, and engineering application of high-performance microalloyed steel. This research result has important academic value and engineering application significance for improving the material properties of microalloyed steel.

## 2. Materials and Methods

**Figure 1:**
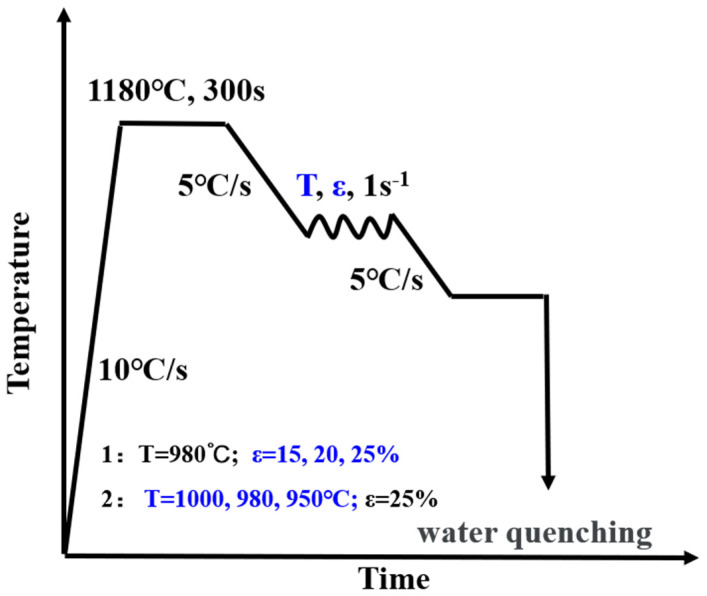
Thermal simulation; laboratory rolling process map (ɛ: deformation).

The thermal simulation of microalloyed steels often employs tools such as JMatPro-API V70 (version V70; Sente Software Ltd., Guildford, UK; for equilibrium phase prediction) and finite element models (for strain–stress coupling). However, these methods may overlook the synergistic effects of Nb dissolution–precipitation kinetics and dynamic recrystallization. To address this, we used Thermo-Calc thermodynamic software (version 2023a, Thermo-Calc Software AB, Solna, Sweden) and a Gleeble-3800 thermal simulation tester (Dynamic Systems Inc., Austin, TX, USA) to simulate two experimental steel states at different rolling stages. The experiments were designed to smelt two kinds of experimental steels, whose actual compositions are shown in Table 1, with #1 steel named Nb steel and #2 steel named Nb-V steel. In this work, a 50 kg vacuum induction furnace is used for smelting, and the billet is forged into 40 mm × 400 mm × 60 mm. Specimens of 8 mm diameter and 12 mm long thermal simulation are cut from the billet, and the thermal simulation process is shown in Figure 1 using the Gleeble-3800 thermal simulation tester; the heat preservation temperature is set to be 1180 °C, which simulates the heat preservation of the actual industrial production. The holding temperature was set to 1180 °C to simulate the holding temperature in actual industrial production. Then, the deformation was carried out at different deformation amounts and deformation temperatures to simulate the roughing stage, and then the specimens were removed and quenched until room temperature was reached.

The thermally simulated finished specimens were then split into longitudinal pairs along the centerline, ground and polished with toothpaste and self-cooling water-cooled sets together, and then rust-etched corroded using a mixture of picric acid and detergent (1:1) and 4% nitric alcohol on the original austenite grains and metallurgical organization of the experimental steels [27]. The data were taken with an OLYMPUS GX53 type metallographic microscope (OM; Olympus Corporation, Tokyo, Japan). The ground and polished specimens were deeply corroded in 4% nitric alcohol (China Steel Research Institute Beijing, China) for 1 min, the carbon film was prepared by using EMS150RS-ion sputtering apparatus (Electron Microscopy Sciences, Hatfield, PA, USA), the carbon film was separated from the steel matrix using nitric alcohol, pure alcohol, and pure water, and finally, the carbon film was loaded with Cu mesh (Zhongjing Scientific Research, Beijing, China) for transmission observation [28,29]; the experimental steel precipitates were used in a high-resolution transmission electron microscope (Tecnai-G2F20, Thermo Fisher Scientific, Waltham, MA, USA) for data. The data were collected and statistically analyzed for the size of the precipitated phase particles using Image-Pro software (version V7.0; Media Cybernetics, Bethesda, MD, USA).

## 3. Results

### 3.1. Effect of Nb-V Composite on Recrystallized Grain Size

**Figure 2:**
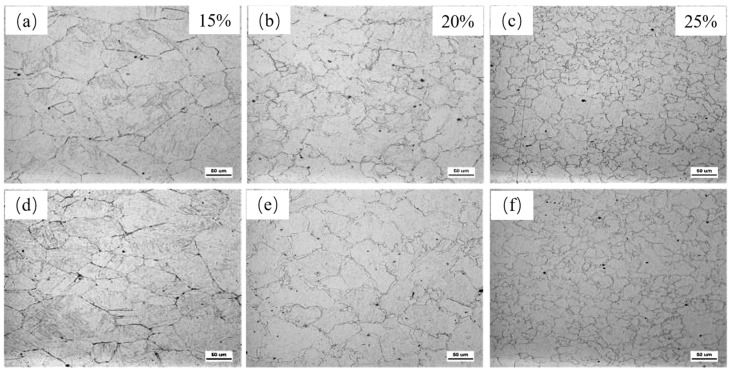
Austenite grains of two test steels at 980 °C with different deformations (**a**–**c**) for Nb Steel and (**d**–**f**) for Nb-V Steel.

Figure 2 illustrates the austenite grain size evolution of the two test steels (Nb and Nb-V steels) after the thermal simulation of the rough rolling process. The austenite histomorphology exhibits significant differences after applying different amounts of deformation at 980 °C, respectively. When the deformation amount was small (15%) (Figure 2a,d), the austenite grains of both test steels showed certain deformation characteristics, elongated along the rolling direction, indicating that the grains nucleated during the deformation process but had not yet completed recrystallization. At this point, the austenite grain size is larger and still retains some of the initial grain characteristics, indicating that the amount of deformation is not sufficient to drive the occurrence of the complete recrystallization process. In contrast, the austenite grain morphology of Nb-V steels is slightly more homogeneous, which may be related to the fact that the presence of the V element facilitates the nucleation process or suppresses the abnormal grain growth.

When the deformation was increased to 25% (Figure 2b,e), complete recrystallization occurred in the austenite of both test steels, the grain size was significantly refined, and the characteristics of grain equal axes became more obvious. This indicates that with the increase in deformation, the deformation storage energy accumulates to a critical level, which promotes the occurrence of dynamic recrystallization. In addition, the austenite grain size of Nb-V steels is slightly smaller than that of Nb steels (Figure 2e vs. Figure 2b), probably due to the effect of V microalloying, which results in stronger inhibition of precipitation relative to the dislocation motion and recrystallization behavior in Nb-V steels, thus increasing the nucleation rate and refining the recrystallized grains.

**Figure 3:**
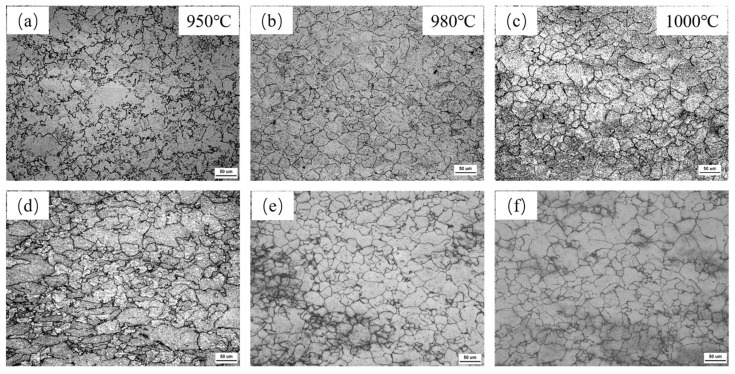
Austenite grains of two test steels at different deformation temperatures for 25% deformation of the test steel (**a**–**c**) for Nb Steel and (**d**–**f**) for Nb-V Steel.

Figure 3 demonstrates the effect of different deformation temperatures on the austenite grain size of Nb and Nb-V steels at 25% deformation. From the figure, it can be observed that the increase in the deformation temperature significantly affects the recrystallization behavior and size evolution of austenitic grains. When the deformation temperature is lower (950 °C) (Figure 3a,d), the austenite grains of both Nb and Nb-V steels are coarser, and obvious deformed grains can still be observed in Nb-V steel (Figure 3d). This indicates that at lower temperatures, the plastic deformation capacity of austenite is limited and dynamic recrystallization is not easy to occur, resulting in some of the grains remaining deformed, while the presence of second-phase particles may further inhibit the nucleation and growth of recrystallization.

When the deformation temperature was increased to 980 °C (Figure 3b,e), the austenite grains of the two experimental steels were obviously refined and began to show local recrystallization characteristics. At this time, due to the increase in temperature, the accumulation of deformation storage energy reached a threshold value conducive to the occurrence of recrystallization, prompting some of the austenitic grains to complete the recrystallization process, thus improving the organization uniformity. However, due to the inhibition of recrystallization by microalloying elements such as Nb and V, the recrystallization process was still incomplete, and the degree of grain refinement was still limited.

When the deformation temperature was further increased to 1000 °C (Figure 3c,f), significant coarsening of austenite grains occurred in both experimental steels. This was mainly due to the fact that higher deformation temperature accelerates the grain growth process, while reducing the inhibition of precipitation relative to recrystallization, thus increasing the austenite grain size. In addition, at higher temperatures, the increase in grain boundary energy also promotes the rapid growth of grains, causing the overall grain size to coarsen.

**Figure 4:**
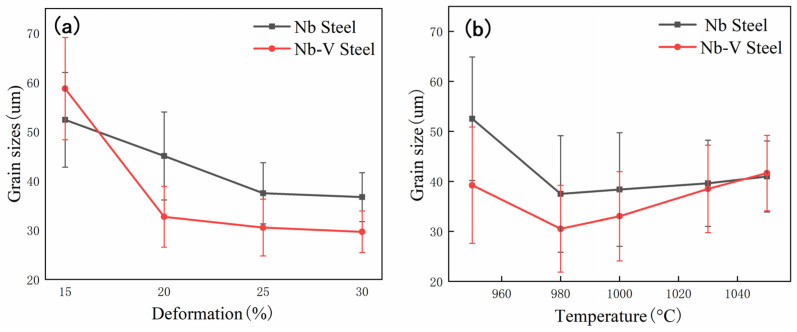
Austenite grain size statistics. (**a**) Test steel at 980 °C temperature with different deformations; (**b**) Test steel at 25% deformation with different temperatures.

Figure 4 further reveals the effects of deformation amount and deformation temperature on the austenite grain size evolution of Nb and Nb-V steels and reflects the mechanism of microalloying elements Nb and V on grain refinement and recrystallization behavior. As can be seen from Figure 4a, the austenite grains of both experimental steels are significantly refined with increasing deformation, but the refinement effect of Nb-V steel is better than that of Nb steel. The main reason for this is the additional inhibition of austenite grain size by the synergistic effect of Nb and V. As a typical grain refining element, the main mechanisms of Nb include the inhibition of recrystallization and promotion of precipitated phase formation. However, an excess of Nb may lead to coarsening or the dissolution of precipitated particles, which reduces its refining effect [30]. When Nb is added in combination with the element V, V promotes the precipitation of carbides and nitrides in austenite, resulting in an increase in the number of precipitated phases and a more diffuse and uniform size. These fine precipitation phases form a significant pinning effect on the austenite grain boundaries, hindering the growth of austenite grains, so that the austenite grain refinement effect of Nb-V steel is better than that of the steel with Nb added alone.

It can be observed from Figure 4b that the austenite grain size of the experimental steels shows a trend of decreasing and then increasing with the increase in the deformation temperature. In the range of 950 °C to 980 °C, the austenite grains are significantly refined, which is mainly attributed to the promotion of recrystallization behavior. In this temperature range, the increase in deformation storage energy promotes the dynamic recrystallization of austenite, and the presence of precipitated phases effectively controls the growth of recrystallized grains, resulting in the refinement of austenite organization. However, when the deformation temperature was further increased to 1050 °C, the austenite recrystallized grains grew significantly, resulting in a subsequent increase in austenite grain size. This phenomenon is mainly related to the acceleration of recrystallization kinetics and dissolution of precipitated phases under high-temperature conditions, which makes the pinning effect of the second phase on the grain boundaries weaker and thus promotes grain growth [31].

It is noteworthy that the degree of grain coarsening in Nb steels is significantly smaller than that in Nb-V steels. This may be related to the differences in the dissolution and precipitation behaviors of Nb and V elements. In the range of 950~1050 °C, element V is basically completely dissolved in austenite, while Nb still exists in the austenite matrix as a precipitated phase. This means that Nb steels are still able to rely on the pinning effect of the second-phase particles to inhibit austenite grain growth at higher temperatures, whereas Nb-V steels have less precipitated phases due to the complete solid solution of elemental V. The inhibition is weakened, and thus the tendency of the austenite grains to grow up is more obvious.

### 3.2. Effect of Nb-V Composite on the Precipitation of the Second Phase

**Figure 5:**
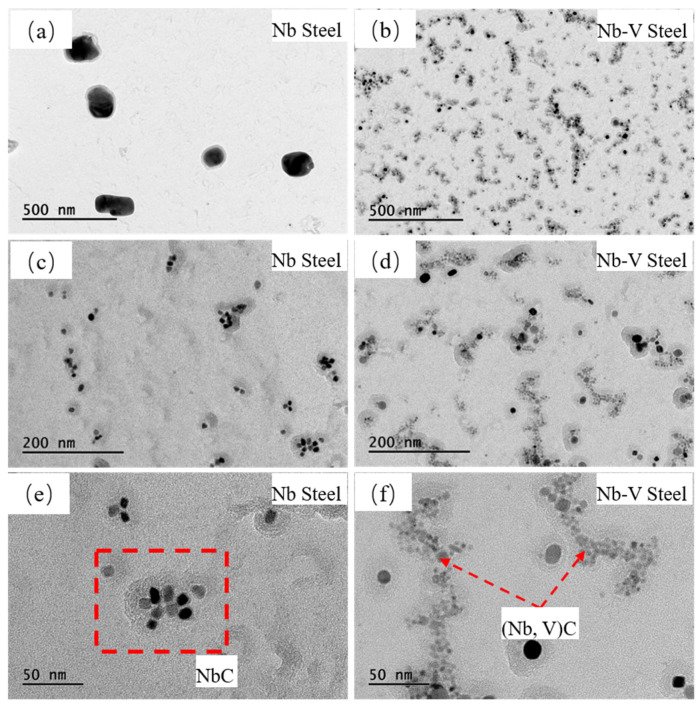
HRTEM images of the morphologies of different scales of precipitates in austenite in experimental steel. (**a**,**c**,**e**) for Nb Steel; (**b**,**d**,**f**) for Nb-V Steel.

To further explain the difference between the deformed austenite grain sizes of the two test steels, we selected a specimen with 980 °C and 25% deformation and made TEM observations of its heart region, as follows:

Figure 5 shows the morphology and distribution of the second phase precipitation at different scales for Nb and Nb-V steels at 980 °C and 30% deformation. Overall, the precipitated phases of high Nb content steels mainly show two different scales of precipitation characteristics, and the large-size precipitated phases are relatively diffuse and uniform. However, under HRTEM (high-resolution transmission electron microscopy) observation, some of the fine precipitated phases of Nb steels showed localized agglomeration (Figure 5c). In contrast, the precipitated phases of Nb-V steels are finer in size and more diffusely distributed (Figure 5b). In addition, in terms of the morphology of the precipitated phases, the precipitated phases in Nb steels are mainly irregularly spherical or square, while the precipitated phases in Nb-V steels are mainly more uniformly square and spherical. It is noteworthy that the spherical precipitated phases in Nb-V steels appear to be characterized by obvious agglomeration or linear distribution, which may be related to the nucleation mechanism of the precipitated phases, of which dislocation line nucleation is one of the most dominant mechanisms [32].

**Figure 6:**
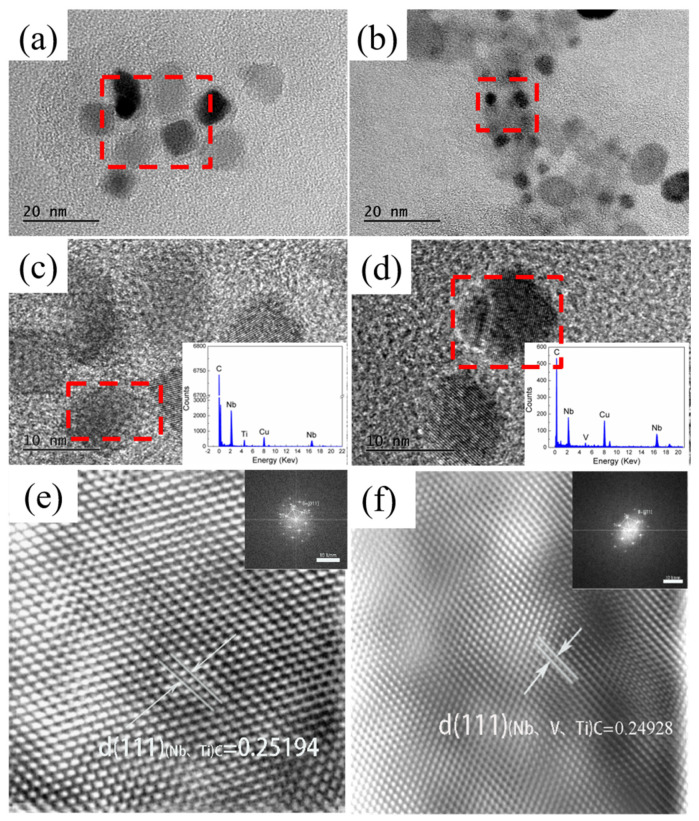
TEM images of the morphology of nanoscale precipitates precipitated in the test steel and high-resolution images of the corresponding particles. (**a**,**c**,**e**) for Nb Steel; (**b**,**d**,**f**) for Nb-V Steel.

In order to further analyze the crystal structure and composition of the precipitated phases, high-resolution characterization of typical precipitated phases was carried out using HRTEM and combined with the GMS 3X software (Gatan, Inc., Pleasanton, CA, USA) for the calculation of crystal plane spacing. It is shown that the precipitated phases of both experimental steels precipitate along the [001] crystal zone axis and are face-centered cubic (FCC) structures. Theoretically, the crystal plane spacing is 0.2581 nm for NbC (111), 0.2493 nm for TiC (111), and 0.2414 nm for VC (111). The (111) crystal plane spacing of the A particles was calculated by the inverse Fourier transform, and it was found to be 0.25194 nm. Together with the EDS spectral analysis (Figure 6c,d), it was confirmed that the precipitation phase of the A particles is (Nb, Ti) C. The precipitation phase of the A particles is (Nb, Ti) C, which is the most important phase of the A particles. Since the experimental steels were all treated with trace Ti (0.01% Ti added) and the N content was lower than 0.004%, it can be assumed that the matrix is almost free of elemental N since the solid solution N content in the matrix was calculated to be lower than 0.0001% at 1250 °C based on the solid solubility product of TiN [33].

In addition, Figure 6d reveals the composite precipitation characteristics of B particles. In the upper left corner of the square precipitation, an obvious spherical precipitation can be observed, forming a typical “cap-shaped” composite precipitation morphology. This phenomenon is consistent with the theory of composite precipitation, i.e., Nb and V elements can be further precipitated on the TiC precipitated particles [26]. The inverse Fourier transform was further used to calculate the (111) crystal plane spacing of the B particles (Figure 6f), which is 0.24928 nm. Combined with the EDS analysis, the main composition of the precipitated phase is (Nb, V, Ti), which further confirms the existence of Nb-V composite precipitation.

**Figure 7:**
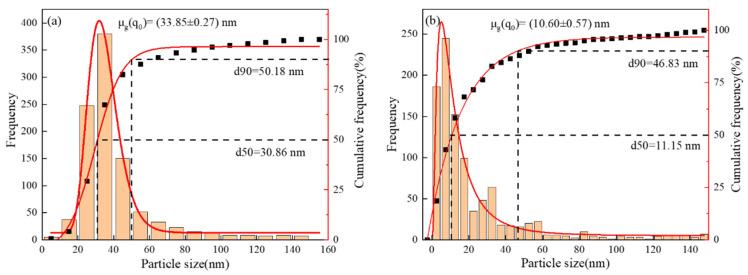
Statistical summary size distribution of precipitated phases for two experimental steels: (**a**) for Nb Steel and (**b**) for Nb-V Steel.

Figure 7 details statistics on the size distribution of 1000 precipitated particles using Image-Pro, showing that the average particle size of the precipitated phase of Nb steel is 33.85 nm, while the average particle size of Nb-V steel is 10.60 nm, indicating that the precipitated phase of Nb-V steel is obviously finer. Further analysis reveals that in the percentage of precipitated particles below 20 nm, Nb-V steels are much higher than Nb steels, and the number of them is also higher. This indicates that the synergistic effect of Nb-V elements can significantly promote the formation of nanoscale precipitated phases and enhance the pinning effect on austenite grain boundaries.

To further quantify the grain size distribution characteristics of the precipitated phases, a normal distribution was fitted to the statistics (Figure 7). The results show that the peak of the precipitated particle size distribution of Nb steels is skewed toward the larger size interval, while the precipitated phase particle size distribution curve of Nb-V steels is significantly shifted toward the smaller size interval, exhibiting finer precipitated phase characteristics. Additionally, the fitting results indicate that the particle size distribution curve of the precipitated phase in Nb-V steel exhibits a narrower peak, suggesting a more uniform and finer distribution of precipitated particles compared to Nb steel, which shows a relatively broader distribution curve. This implies that the second-phase particles in Nb-V steel are more evenly sized and finer in comparison to those in Nb steel.

In addition, from the distribution characteristics, 50% of the precipitated particles of Nb-V steel are in the small size interval (<20 nm), while the precipitated phase of Nb steel, d50 = 30.86 nm, is much larger than that of the Nb-V composite experimental steel, which suggests that Nb-V composite additions are able to more efficiently promote the formation of fine precipitated phases, while reducing the generation of larger precipitated phases. This may be closely related to the changes in the precipitation kinetics after Nb, V composite addition; the Nb-V precipitation phase has a higher nucleation rate, and in the nucleation process, it effectively inhibits the growth of the coarse precipitation phase and ultimately the formation of a more diffuse and uniform precipitation structure.

## 4. Discussion

### 4.1. Thermodynamic Calculations for Experimental Steels

First, Thermo-Calc thermodynamic software was used to calculate the equilibrium phase diagrams of the test steels and to analyze the relationship curves of the content of each phase and the alloying element components in the second phase with the change in temperature. The results of this calculation provide a theoretical basis for the prediction and calculation of the subsequent second-phase precipitation behavior and also provide an important reference for the exploration of the second-phase precipitation mechanism under different process conditions.

**Figure 8:**
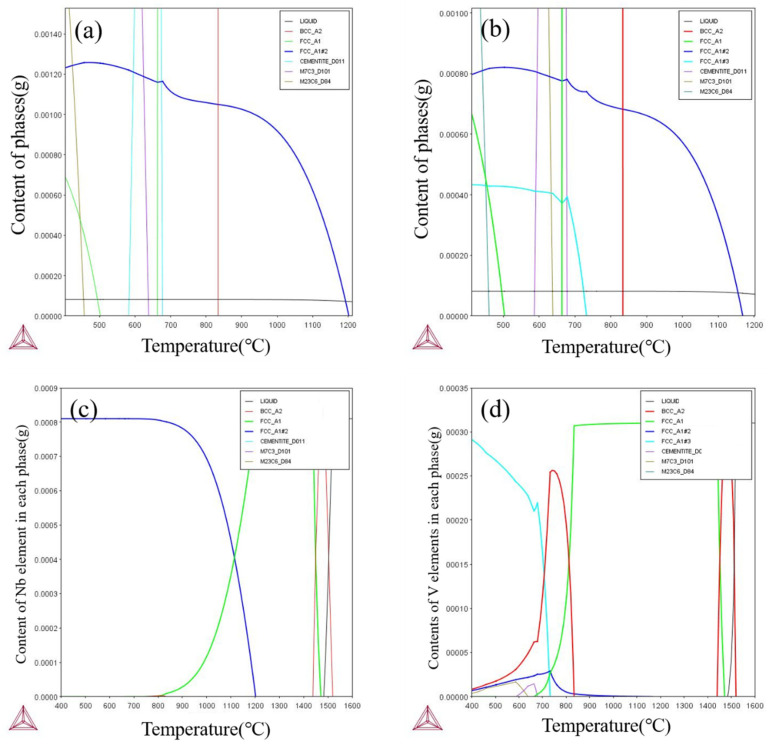
Thermodynamic calculations of experimental steels. (**a**,**c**) for Nb Steel and (**b**,**d**) for Nb-V Steel.

Figure 8a,b demonstrates the temperature dependence of each phase content in high Nb and Nb-V steels, respectively, where FCC_A1#2 represents the MC-type second phase. The results show that the MC second phase starts to precipitate below 1200 °C, with the fastest precipitation rate around 980 °C, and the trend of precipitation slows down below 900 °C. This is mainly due to the dissolution of the MC phase in the high temperature stage, and the precipitation amount increases abruptly near 980 °C due to the decrease in solubility; then, the precipitation rate slows down due to the decrease in precipitable elements.

Unlike high Nb steels, Nb-V steels also show the FCC_A1#3 precipitation phase below 900 °C, which is due to the addition of the V element to change the precipitation behavior. The higher solubility of V in austenite makes it difficult to precipitate at high temperature, and instead, the second phase of nano-sized MC is precipitated in ferrite, which enhances the precipitation strengthening effect. This allows Nb-V steels to still have precipitation strengthening potential at the low temperature stage, which affects the final organization and properties.

The elemental composition of the MC second phase is further analyzed in Figure 8c, where the MC phase in high-Nb steels is mainly (Nb, Ti)C, which has a high thermal stability and strengthening effect, whereas the precipitated phase of Nb-V steels may contain Nb, V, and Ti at the same time, which makes the precipitated phase finer and more uniform, and contributes to the enhancement of material strength and toughness. This suggests that the Nb-V alloying strategy can effectively optimize the precipitation behavior, which provides an important basis for tissue modulation and property enhancement.

**Figure 9:**
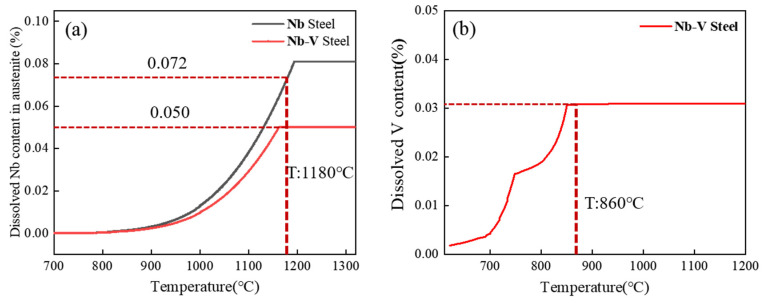
Thermodynamic calculations of two experimental steels: (**a**) for Nb Steel and (**b**) for Nb-V Steel.

The solid solution behavior of Nb and V microalloying elements in the two kinds of test steels at different temperatures was calculated using Thermo-Calc thermodynamic software, and the results are shown in Figure 9. As can be seen in Figure 9a, the solid solution Nb element content in Nb and Nb-V steels at 1180 °C is 0.072% and 0.05%, respectively, which indicates that most of the Nb has been Nb solidly dissolved into the austenite, while the V element is completely solidly dissolved in the austenite (Figure 9b).

This solid solution behavior corresponds to the results of the austenite original grain size (Figure 4b), indicating that the microalloying elements solidly dissolved in the austenite at elevated temperatures also play a significant role in inhibiting grain growth. Specifically, the precipitated phase of Nb effectively hinders the austenite grain growth by pinning the grain boundaries, whereas V has a weaker inhibitory effect on grain growth at this temperature due to its complete solid solution. Therefore, the presence of precipitated phases is a key factor in controlling the austenite grain size.

### 4.2. Dynamics Calculations for Experimental Steels

Microalloying elements in test steels exist mainly in two forms: one part is solidly dissolved in the iron matrix and the other part precipitates as a second phase. Under specific conditions, the two forms can reach equilibrium, and the corresponding microalloying element contents are relatively stable. Therefore, in this study, the precipitation behavior of the second phase of composite microalloying is theoretically calculated and based on the following assumptions:

(1) All Ti elements in the steel are precipitated in the form of large TiN particles, and the influence of N elements on the precipitation of (Nb, V) C is not considered;

(2) NbC and VC have a NaCl structure, can be completely solid solutions, and meet the ideal stoichiometric ratio, forming a composite precipitation phase with the chemical formula of Nb_x_V_1-x_C;

(3) (Nb, V) C nucleates preferentially on the dislocation line and its nucleation rate decays rapidly to zero;

(4) The morphology of the precipitated MC phase in austenite is approximately spherical, and the effect of elastic strain energy is not taken into account when calculating the free energy change in individual nuclei;

(5) Since the test steels were treated with micro-titanium, the effect of Ti on the composite precipitation is negligible.

The nucleation of microalloyed carbides (e.g., (Nb, V) C) exhibits strong spatial heterogeneity due to preferential nucleation at grain boundaries (GBs). Thermodynamic: GBs provide lower activation energy for nucleation compared to dislocation lines (DLs). Kinetic transition: As GB regions become solute-depleted (Nb/V concentration < at 980 °C [34,35]), nucleation shifts to DLs where residual solute accumulates via pipe diffusion. This transition creates a non-uniform precipitation landscape, characterized by coarse GB precipitates and finer DL precipitates. Decaying GB nucleation dominance: The GB nucleation rate decays exponentially with solute depletion time.

It is assumed that the initial contents of the elements Nb, V, and C in the test steel are the contents of Nb, V and C, respectively. The solid solution of each element in the austenite of the test steel is expressed as [*Nb*], [*V*], and [*C*], which can be obtained based on the previous assumptions, as follows:

(1) $$\mathrm{lg}\frac{\left[Nb]\cdot [C\right]}{x}=2.96-7510/T$$

(2) $$\mathrm{lg}\frac{\left[V]\cdot [C\right]}{1-x}=6.72-9500/T$$

(3) $$\frac{0.05-[Nb]}{0.055-[C]}=\frac{92.9064}{12.011\cdot x}$$

(4) $$\frac{0.73-\left[V\right]}{0.055-\left[C\right]}=\frac{50.9414}{12.011\cdot \left(1-x\right)}$$

[*X*] is the amount of element X solidly dissolved in austenite, *x* represents the partition coefficient of Nb-V, and T represents the temperature. The corresponding solid solubility product equation for (Nb, V) C in austenite can be derived as

(5) $$\mathrm{lg}\{{\left[Nb\right]}^{x}\cdot {\left[V\right]}^{\left(1-x\right)}C\}=2.96x+6.72\left(1-x\right)+x\mathrm{lg}x+\left(1-x\right)\mathrm{lg}\left(1-x\right)-\frac{\left\{7510x+9500\left(1-x\right)\right\}}{T}$$

By substituting the values of [*Nb*], [*V*], and [*C*] at different calorimetric temperatures, the equilibrium solid solution and equilibrium coefficients *x* for each element can be obtained by solving the equilibrium solid solution and equilibrium coefficients x for each element at the determined temperatures, as shown in Table 2 (*x* is taken to be 1 for Nb steels and *x* is taken to be 0 for V steels).

When the homogenization temperature is lower than 1279 °C, then the free energy of phase transition for precipitation of (Nb, V) C at different precipitation temperatures T is

(6) $$\Delta G_{m}=-19.1446B\prime +19.1446T\left\{A\prime -\mathrm{lg}\left({\left[Nb\right]}^{x}{\,}_{H}\times {\left[V\right]}^{\left(1-x\right)}{\,}_{H}\times {\left[C\right]}_{H}\right)\right\}$$

If x does not vary much in the above temperature range, the calculation can only be made using the approximate value of x. Moreover, the specific interfacial energies of NbC and VC at each temperature are given as follows:

(7) $$\sigma_{NbC}=1.3435-0.6054\times 10^{-3}T$$

(8) $$\sigma_{VC}=1.1292-0.5088\times 10^{-3}T$$

The specific interfacial energy between (Nb, V) C and austenite can be obtained using linear interpolation for different values of x, as follows:

(9) $$\sigma_{Nb_{x}V_{1-x}C}=\left[1.3435x+1.1292\left(1-x\right)\right]-\left[0.6054x+0.5088\left(1-x\right)\right]\times 10^{-3}T$$

At this point, if the variation in x in the temperature range under consideration is not large, x is essentially constant and has a relatively small impact error on the specific interfacial energy estimation, with a relative change in specific interfacial energy of only 0.1% when the x value changes by 0.03. Therefore, the approximate value of x can be used to calculate the specific interfacial energy.

For a dislocation line nucleus, the free energy change to form a spherical nucleus embryo of diameter ∆G is as follows:

(10) $$\Delta G=\frac{1}{4}\pi d^{3}\Delta G_{v}+\pi d\sigma -AInd+C$$

where *C* is a constant independent of *d*. or $A=\frac{Gb^{2}}{\left[4\pi \left(1-v\right)\right]}\;$ and $A=Gb^{2}/(4\pi )$, *G* is the shear elastic modulus, *v* is the Poisson’s ratio, and b is the dislocation Bergs vector. When $\frac{\sigma ∆G_{d}}{\theta d}=0$, the critical nucleation size of microalloyed carbides can be obtained as $d_{d}^{*}$, as follows:

(11) dd∗=−2σΔGv1+1+β12

(12) $$\beta =\frac{A\Delta G_{v}}{2\pi \sigma^{2}}$$

critical form nuclear work $∆G_{d}^{*}$:

(13) Gd∗=ΔGdd∗−ΔGd0=16πσ33ΔGV21+β32=1+β3/2ΔG∗

*ΔG*∗ represents the energy barrier to form a stable precipitate nucleus. In Nb-V steels, chemical synergy of Nb-V co-precipitation occurs, lowering the interfacial energy by 15–20% compared to pure NbC. The model quantifies why Nb-V accelerates nucleation but restrains growth, aligning with the observed high density of nano-precipitates. The volume change law of the precipitated phase with time change rule is $l\pi r^{2}=\pi l\lambda^{2}Dt$. Integrating over the time from 0 to t yields the kinetic equations for the case where the precipitated phase nucleates on the dislocation line and the nucleation rate shrinks to zero, as follows:

(14) $$X=1-\exp[-\int_{0}^{t}I_{d}\pi l\lambda^{2}Dtd\tau ]=1-\exp(-\pi I_{d}\tau_{1}l\lambda^{2}Dt)$$

It follows that when (Nb, V) C nucleates on the dislocation line and decays rapidly to zero, *n* = 1, as follows:

(15) $$\begin{array}{l} B_{da}=\pi I_{d}\tau_{1}l\lambda^{2}D\\ =\pi^{2}K\rho b^{2}D_{0}l\lambda^{2}d_{d}^{*2}\exp(-\frac{{\left(1+\beta \right)}^{3/2}\Delta G^{*}+Q_{d}+Q}{kT}) \end{array}$$

Substituting into Equation (14):

$$\mathrm{lg}t_{0.05da}=-1.28994-\mathrm{lg}\mathrm{lg}\frac{15\pi \tau_{1d}\rho b^{2}l\lambda^{2}}{8D_{0}^{1/2}\cdot {\left[\frac{2(c_{0}-c_{M})}{c_{N}-c_{M}}\right]}^{3/2}}-2\mathrm{lg}d_{d}^{*}+\frac{1}{\ln10}\times \frac{{\left(1+\beta \right)}^{3/2}\Delta G^{*}+\frac{5}{3}Q}{kT}$$

(16) $$\mathrm{lg}t_{0da}=-\left[\mathrm{lg}c+\mathrm{lg}\frac{15\pi \tau_{1d}\rho b^{2}l\lambda^{2}}{8D_{0}^{1/2}\cdot {\left[\frac{2(c_{0}-c_{M})}{c_{N}-c_{M}}\right]}^{3/2}}\right]$$

where *t*_0*d*_ is a temperature independent constant. Therefore, we obtain the following:

(17) $$\mathrm{lg}\frac{t_{0.05da}}{t_{0da}}=-1.28994-2\mathrm{lg}d_{d}^{*}+\frac{1}{\ln10}\times \frac{{\left(1+\beta \right)}^{3/2}\Delta G^{*}+\frac{5}{3}Q}{kT}$$

d_d_^*^ represents the critical kernel size; *β* is the nucleation factor on the dislocation line; ΔG* is the critical kernel energy; *Q* is the activation energies; *k^−1^* is the Linear expansion coefficient; and *T* is the temperature. Based on relevant thermodynamic data and literature references, we obtained the linear expansion coefficients of NbC, VC, and the activation energies of Ti, V, and Mo. The results are shown in Table 3 and Table 4.

**Figure 10:**
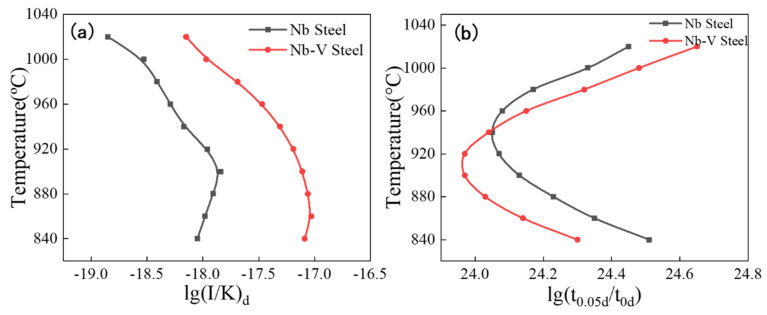
NrT and PTT curves of NbC and (Nb, V) C precipitated in austenite (**a**) for NrT and (**b**) for PTT.

The PTT and NrT curves for precipitation of (Nb, V)C in austenite were calculated from Table 5 and are shown in Figure 10. Figure 10a shows the NrT curves of precipitation in austenite for Nb and Nb-V steels with nose point temperatures of 1000 °C and 960 °C, respectively, and the NrT curve of Nb-V steel is always located in the upper right corner of Nb steel, indicating that the nucleation rate of Nb-V steel is always higher than that of Nb steel. This indicates that the addition of the V element promotes the second phase nucleation in austenite of Nb-V steel and accelerates the kinetic process of precipitation.

Figure 10b shows the PTT curves of precipitation in austenite for the experimental steels with nose point temperatures of 940 °C and 920 °C, respectively, with small differences, but there is an obvious crossover in the PTT curves. This crossover phenomenon is mainly controlled by the combination of two independent factors: at higher temperatures, the chemical drive plays a dominant role, and the experimental steel with higher Nb content has a greater chemical drive at this time due to the fact that the V element is basically solidly dissolved in the austenite [36]; whereas, at lower temperatures, the atomic diffusion dominates the precipitation process, and the Nb and V elements are precipitated at the same time and promote each other [37]. In the medium temperature interval, the combined effect of the two mechanisms reaches a maximum, forming a typical C-shaped PTT curve. In addition, the PTT curves of Nb-V steels are shifted to the lower left compared to Nb steels, indicating that the time required for precipitation is shorter at the nose point temperature. This result further demonstrates the promoting effect of element V on the precipitation of Nb in austenite, which leads to a faster precipitation rate of the second phase in Nb-V steels and thus improves the precipitation strengthening effect. This means that in actual industrial production processes, Nb-V compounding can effectively improve the strength of our low-carbon microalloyed steel, reduce the addition of alloying elements, maximize the potential of microalloying elements, and effectively control costs.

The experimental results show that there is a significant difference between the austenite recrystallization behavior of Nb and Nb-V steels under different deformation conditions. As can be seen from Figure 4, with the increase in deformation, the austenite of the two experimental steels undergoes recrystallization, and the grains are all significantly refined, but the refinement effect of Nb-V steel is superior, which is mainly attributed to the inhibition effect of grain boundary migration by the precipitation of more second phase Nb-V steel. Combined with the precipitation phase statistics in Figure 6, it can be seen that the precipitation phase particle size of Nb-V steel is smaller and more numerous, especially the proportion of nanoscale precipitation phases below 100 nm, which is significantly higher than that of Nb steel, and these diffusely precipitated particles play a stronger pinning effect on the austenite grains, which can effectively impede the growth of the austenite grains and improve their recrystallization refinement degree.

**Figure 11:**
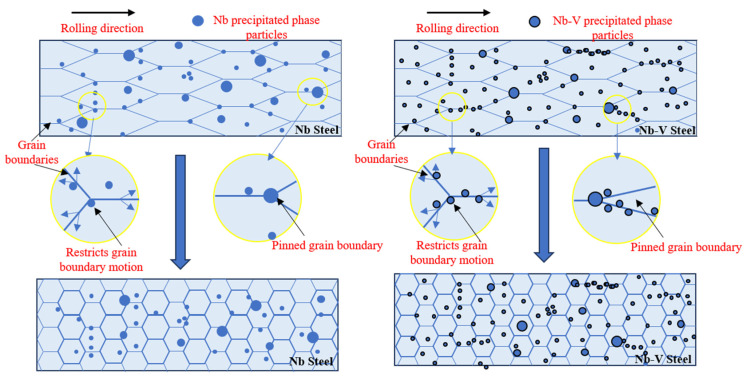
Schematic diagram of the second phase granular precipitation pinned grain boundaries.

In addition, from the thermodynamic calculations (Figure 7) and kinetic analysis (Figure 10), it can be seen that in the austenite phase region, the Nb element precipitates in the form of the MC phase, while the V element is mainly in solid solution at high temperatures and then gradually precipitates at low temperatures and forms a composite precipitation phase with NbC (Nb, V) C. The results of the thermodynamic calculations show that the precipitation temperature of the MC phase is lower, the rate of precipitation is faster, and the nucleation rate of it is higher than that of the Nb-V steel (NrT curve). The NrT curve is higher than that of Nb steel, indicating that the addition of the V element effectively promotes the precipitation kinetic process. The left shift of the PTT curve indicates that the precipitation time of Nb-V steels is shorter, which is conducive to the formation of more diffuse precipitated phases during the deformation process and further enhances the effect of austenite grain refinement. To further illustrate the effect of Nb-V composite on the precipitation and recrystallization of the second phase, we analyzed its schematic diagram Figure 11.

In conclusion, as shown in the schematic diagram in Figure 11, there is an obvious interaction between austenite recrystallization and the precipitation of the second phase, and the precipitated (Nb, V) C phase in Nb-V steels is able to nail the austenite grain boundaries more effectively at high temperatures to inhibit its growth, while further precipitating fine particles at lower temperatures to enhance the restraining effect on the austenite grains. This synergistic effect of precipitation and recrystallization enables the formation of finer and more homogeneous austenite grains in Nb-V steels during hot deformation, which provides an important basis for the subsequent organization and property optimization.

## 5. Conclusions

In this study, the rough rolling stage of traditional industry is simulated through thermal simulation experiments; the austenite grain size of the test steel and the deformation amount of the rough rolling temperature are established in connection with each other, the influence of Nb-V microalloying on the precipitation of the second-phase particles is discussed in depth through the thermodynamic and kinetic calculations, and finally, the connection between the Nb-V microalloying, the precipitation of the second-phase particles, and the austenite recrystallization behavior is established, and the following conclusions are obtained:

(1) The deformation amount and deformation temperature have significant effects on austenite grain size and recrystallization behavior. At the roughing temperature of 980 °C, as the roughing deformation increases from 15% to 30%, the austenite size of Nb steel decreases from 52.4 μm to 36.71 μm, that of Nb-V steel decreases from 58.74 to 29.67 μm, and the degree of refinement of Nb-V steel is larger than that of Nb steel. In the roughing deformation of 20%, with the roughing temperature increased from 950 °C to 1050 °C, the austenite size of Nb steel first reduced from 52.54 μm to 37.49 μm and then increased to 40.98 μm, and Nb-V steel at 39.23 μm reduced to 30.52 μm and then increased to 41.66 μm;

(2) Significant differences in the temperature range, morphology, and size distribution of the second phase precipitated from Nb and Nb-V steels were observed by carbon film precipitation transmission experiments. Through high-resolution characterization to further verify the Nb-V composite microalloyed steel precipitation phase particles for (Nb, V) C, while the Nb steel precipitation phase is only NbC on Nb steel, Nb-V steel carbon film precipitation experiments and phase analysis experiments found the second phase of Nb steel for NbC, with an average particle size of the particles for 33.85 nm. Nb-V steel in the second phase has particles of an average size of 10.60 nm; the particle number is greater, and the distribution is more diffuse;

(3) Through thermodynamic and kinetic calculations, the NrT and PTT curves of Nb and Nb-V experimental steels are calculated, and the temperature of the nose point of the PTT curve of Nb steel is 940 °C. The Nb-V steel PTT curve of the nose point temperature is 920 °C. The Nb-V steel NrT curve is in the upper right of the Nb steel, and the PTT curve in the lower left. For Nb-V steel, the second phase of the second phase precipitation time is shortened to improve the rate of precipitation kinetics, indicating that the Nb-V composite has a promotional effect on the precipitation of its second phase;

(4) Through thermal simulation experiments, it is learned that the Nb-V composite, from the thermodynamic and kinetic point of view, are conducive to the precipitation of the second-phase particles and rough rolling process precipitation of the second-phase particles by hindering the movement of grain boundaries on the recrystallization of the behavior of the obvious limitations. From the experimental results, the second-phase grains of the obstruction are significantly greater than the drag effect of the solid solution atoms of Nb, V;

(5) While this study clarifies the synergistic effects of Nb-V microalloying on precipitation kinetics and recrystallization suppression during rough rolling, several critical challenges remain for industrial implementation. 1: Multi-element interaction mechanisms. 2: Dynamic process window optimization. 3: Sustainable processing routes.

## Figures and Tables

**Table 1:** Chemical composition of experimental steel (mass fraction, %).

Steels	C	Mn	Si	Ni	Mo	Cr	Cu	Nb	V	Ti
Nb Steel	0.054	1.73	0.214	0.103	0.155	0.262	0.095	0.080	/	0.012
Nb-V Steel	0.055	1.73	0.221	0.104	0.156	0.262	0.096	0.050	0.031	0.010

**Table 2:** Variation in the value of X with temperature.

Temperature (°C)	940	960	980	1000	1020	1040	1060	1080	1100
X	0.886	0.891	0.895	0.900	0.903	0.909	0.916	0.927	0.933

**Table 3:** Lattice constants and linear expansion coefficients of NbC, VC at room temperature.

Carbide	Lattice Constant/nm	Linear Expansion Coefficient/k^−1^
NbC	0.4469	7.02 × 10^−6^
VC	0.4182	8.29 × 10^−6^

**Table 4:** Activation energies of Ti, V, and Mo in g and a matrix [26].

Element	Q_g_/kJ	Q_a_/kJ
Nb	267	252
V	264	241

**Table 5:** Calculation results of the nucleation parameters of Nb steel and Nb-V steel in austenite.

Temperature/°C	Nb Steel			Nb-V Steel		
	d*d/nm	lg(I/K)_d_	lg(t_0.05_/t_0_)	d*d/nm	lg(I/K)_d_	lg(t_0.05_/t_0_)
940	0.59	−18.22	24.51	0.58	−17.09	24.3
960	0.60	−18.13	24.35	0.59	−17.03	24.14
980	0.62	−17.95	24.23	0.60	−17.06	24.03
1000	0.65	−17.84	24.13	0.61	−17.11	23.97
1020	0.67	−17.96	24.07	0.63	−17.19	23.97
1040	0.70	−18.17	24.05	0.64	−17.31	24.04
1060	0.71	−18.29	24.08	0.66	−17.47	24.25
1080	0.73	−18.41	24.17	0.68	−17.69	24.42
1100	0.75	−18.53	24.33	0.69	−17.97	24.68
1120	0.78	−18.85	24.45	0.71	−18.15	25.05

## Data Availability

The original contributions presented in this study are included in the article. Further inquiries can be directed to the corresponding author.
